# Mineralocorticoid receptor antagonists use in patients with heart failure and impaired renal function

**DOI:** 10.1371/journal.pone.0258949

**Published:** 2021-10-28

**Authors:** Anna Jonsson Holmdahl, Helena Norberg, Fredrik Valham, Ellinor Bergdahl, Krister Lindmark

**Affiliations:** 1 Department of Public Health and Clinical Medicine, Umeå University, Umeå, Sweden; 2 Department of Integrative Medical Biology, Umeå University, Umeå, Sweden; Scuola Superiore Sant’Anna, ITALY

## Abstract

**Aims:**

Impaired renal function is a major contributor to the low proportion of mineralocorticoid receptor antagonist (MRA) treatment in patients with heart failure with reduced ejection fraction (HFrEF). Our aims were to investigate the impact of MRA treatment on all-cause mortality and worsening renal function (WRF) in patients with HFrEF and moderately impaired renal function.

**Methods:**

Retrospective data between 2010–2018 on HFrEF patients from a single-centre hospital with estimated glomerular renal function (eGFR) < 60 ml/min/1.73 m^2^ were analysed. WRF was defined as a decline of by eGFR ≥ 20%.

**Results:**

416 patients were included, 131 patients on MRA and 285 without MRA, mean age was 77 years (SD ± 9) and 82 years (SD ± 9), respectively. Median follow-up was 2 years. 128 patients (32%) experienced WRF, 25% in the MRA group and 30% in patients without MRA (p = 0.293). In multivariable analysis, hospitalization for heart failure and systolic blood pressure were associated with WRF (p = 0.015 and p = <0.001), but not use of MRA (p = 0.421). MRA treatment had no impact on the risk of adjusted all-cause mortality (HR 0.93; 95% CI, 0.66–1.32 p = 0.685). WRF was associated with increased adjusted risk of all-cause mortality (HR 1.43; 95% CI, 1.07–1.89 p = 0.014). Use of MRA did not increase the adjusted overall risk of mortality even when experiencing WRF (HR 1.15; 95% CI, 0.81–1.63 p = 0.422).

**Conclusion:**

In this cohort of elderly HFrEF patients with moderately impaired renal function, MRA did not increase risk for WRF or all-cause mortality.

## Introduction

Moderately impaired renal function is a common reason for not initiating treatment with Mineralocorticoid receptor antagonists (MRA) in clinical practice in patients with heart failure with reduced ejection fraction (HFrEF) [1, 2], due to the fear of worsening renal function (WRF) and hyperkalemia. WRF is commonly defined as an increase in serum creatinine (s-creatinine) of more than 26.5 μmol/l or over 25% or as a decrease in eGFR over 20% and is an WRF independent predictor of worse outcome in patients with HFrEF [3–6].

In landmark trials, MRA in addition to angiotensin-converting enzyme inhibitors (ACEI)/angiotensin receptor blocker (ARB) and beta blockers (BB) has proven to decrease mortality and hospitalization rates for patients with HFrEF [7–10]. A substantial underuse of particularly MRA has been reported, as only about 33–42% of all eligible HFrEF patients are treated with MRA [1, 10–13]. Reasons for undertreatment with MRA are mainly lower estimated glomerular filtration rate (eGFR) (<60 ml/min/1.73 m^2^), non-specialist care, milder New York Heart Association (NYHA) functional class and no use of other heart failure therapy [1, 2, 14]. Furthermore, use of evidence-based therapy is lower in patients with higher risk of mortality, suggesting a “risk-treatment paradox”. A common explanation is concern for complications due to WRF [15]. According to guidelines, if eGFR decreases below 30 ml/min/1.73 m^2^ or potassium increases to >5.5 mmol/L during MRA use, the dose should be reduced by 50%. If eGFR decreases below 20 ml/min/1.73 m^2^ or potassium increases to over 6.0 mmol/L that MRA should be immediate discontinued [10].

Since impaired renal function is a major contributor to the low proportion of MRA treatment in patients with HFrEF, our aims were to investigate all-cause mortality and factors associated with WRF in patients with HFrEF and moderately impaired renal function that are treated with MRA compared to patients not treated with MRA.

## Methods

### Ethical approval

This study complies with the Declaration of Helsinki. The Regional Ethical Review Board in Umeå, Sweden has approved this study (registration number 2015/419–31). Patients’ medical records are protected by confidentiality by the Public Access to Information and the Secrecy Act but can be available for research purposes after an approval by an Ethical Review Board. We did not obtain informed consent from the included patients, which was waived by the Ethical Review Board.

### Study design and patient population

This was a retrospective, observational, single-centre study.

Medical records were screened for all patients who received a diagnoses of heart failure (International Classification of Diseases codes I50.X, I42.0, I42.6, I42.7, I42.9, I11.0, I13.0 and I13.2) who had at least one contact with Heart Centre or Department of medicine at Umeå University Hospital Sweden between 2010 and 2018. Both prevalent and incident patients were included. All patients with Ejection Fraction (EF) ≤ 40% and eGFR <60 ml/min/1.73 m^2^ were included. We excluded all patients who died before January 1, 2016.

### Data collection

We manually collected data from the medical records regarding medical therapy, laboratory data, clinical-, echocardiogram- and electrocardiography parameter. Renal function was classified into CKD classes by eGFR, with CKD 3 representing eGFR 30–59, CKD 4 representing eGFR 15–29 and CKD 5 eGFR <15 [16]. Patients were included from January 1, 2010, until March 20, 2018. There were two data collection points. The index collection point for incident cases were the time of first heart failure diagnosis, and for prevalent cases, who were diagnosed before January 1 2010, the journal entry closest to this date. The follow-up data collection point was the journal entry that was closest to the end of the data collection period. Data on mortality were collected from January 1, 2010, until May 07, 2020.

In patients that discontinued MRA, medical records were scrutinized to find how many patients that discontinued MRA treatment due to renal dysfunction.

### Outcomes

The investigated outcomes in this study were decline in renal function, WRF and all-cause mortality.

### Definition of WRF

eGFR was computed according to the revised Lund-Malmö equation [17]. We defined WRF as a decline of eGFR of at least 20% or more between index and follow-up [18].

### Statistical analysis

All analyses were performed in IBM SPSS Statistics version 25. The two-tailed significance level was set at p<0.05. Continuous variables are expressed as mean and standard deviation (SD) when normal distributed and as medians with inter-quartile range (IQR) when not normal distributed. Categorical variables are presented as frequencies (percentage). Odds ratio (OR) and hazard ratio (HR) are presented as estimate and 95% confidence interval (CI). Comparison of characteristics and differences in renal function was carried out with the Pearson *χ*2 test for categorical variables and Fisher exact test when appropriate. Students *t* test were used for continuous values with normal distribution and Mann-Whitney U-test when not normal distributed.

Multivariable logistic regression was used to assess factors associated with WRF. All covariates were included in the analysis simultaneously. We defined WRF as a categorical value, as present or not present, with a cut-off at 20%.

Kaplan-Meier estimator were used to construct cumulative survival groups for the On MRA and No MRA groups. The primary comparison between the two groups were based on the log-rank test. Association of all-cause mortality and MRA use was assessed with the Cox proportional hazard model. We performed the analyse adjusting for the following covariates: sex, age, index eGFR and WRF. Assumptions of proportionality of hazard were verified by log-log plots.

## Results

### Patient characteristics

**Fig 1:**
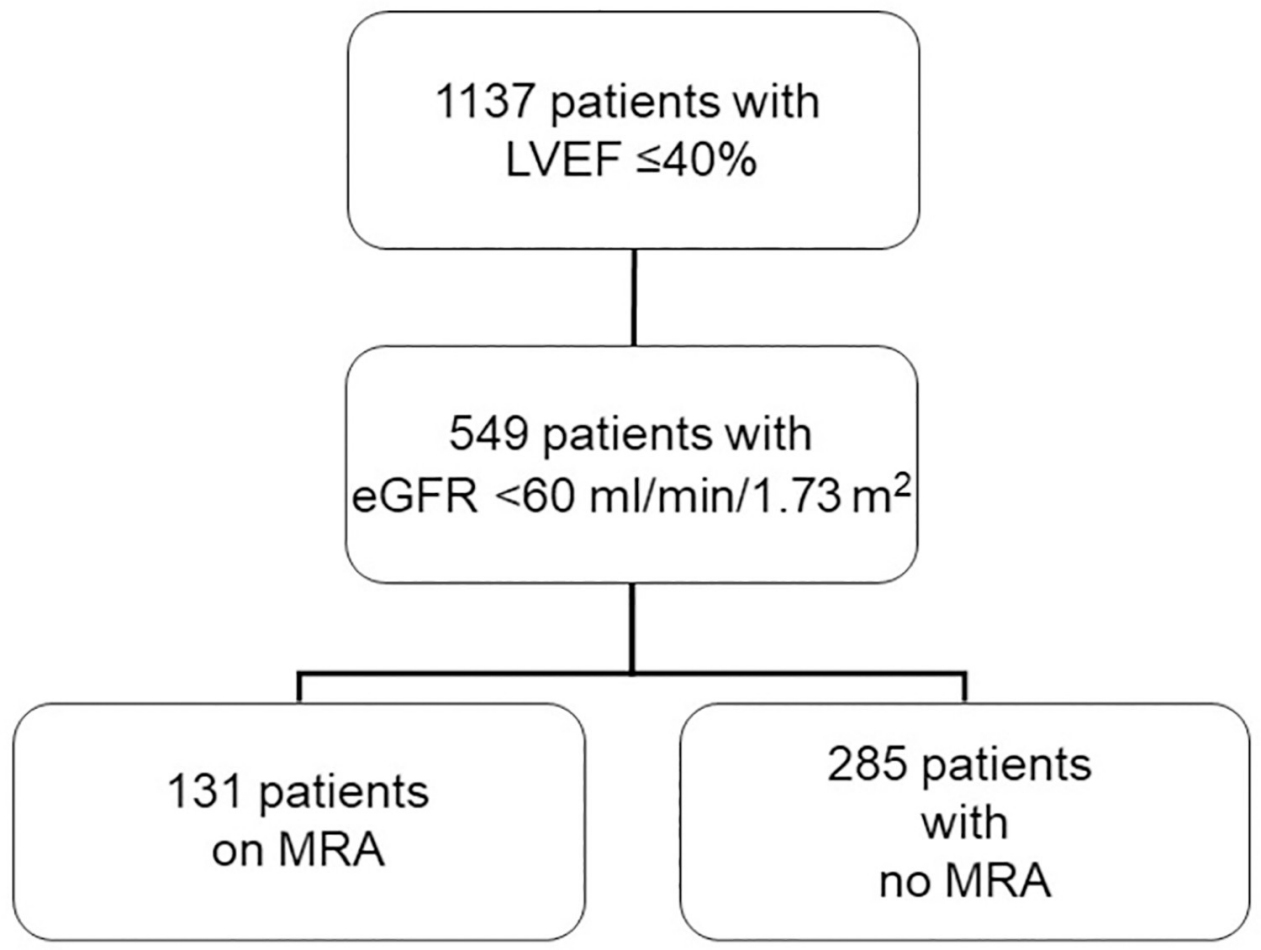
Patient selection flow chart.

Out of a total of 4449 patients with heart failure, 2955 patients were alive January 1, 2019. We excluded 17 patients that died within 1 month after heart failure diagnosis. 1137 patients (26%) had LVEF ≤40%. 549 (48%) had eGFR < 60 ml/min/1.73 m^2^. Of the 549 patients we compared the group who had MRA during both index and follow-up, the On MRA group (N = 131) with the group without MRA at both index and follow-up, the No MRA group (N = 285) without MRA at index and follow-up. Hence, a total of 416 patients were included in the final analysis (Fig 1).

Characteristics at index showed that patients On MRA and had about 5 months shorter follow up time between the two s-creatinine values and the median follow-up was about 2 years. The On MRA were followed 649 days and the No MRA were followed 799 days (p = 0.048). When stratified, 42% were followed ≤ 1 year, 33% were followed 1–3 years and 26% were followed 3–6 years.17% in On MRA and 25% in No MRA were prevalent patients where the index data collection was January 1, 2010 (p = 0.065). A majority of all patients were in CKD class 3, 127 (97%) in On MRA and 225 (79%) in No MRA (p <0.001), although more patients in No MRA were in lower CKD-classes.

Noteworthy, patients were equally distributed by treatment with ACEI/ARB, BB, female sex and comorbidities. Index LVEF was lower in the On MRA group. (Table 1).

**Table 1:** Characteristics of patients according to MRA use.

Characteristic	On MRA (n = 131)	No MRA (n = 285)	*p*
**Female sex, n (%)**	47 (36)	101 (35)	0.931
**Age, y**	77 ± (9)	82 ± (9)	<0.001
**EF, %**	33 ± (9)	35 ± (9)	0.025
**Medical history, n (%)**			
** Diabetes**	37 (28)	80 (28)	0.971
** Hypertension**	88 (67)	213 (75)	0.109
** Coronary artery disease**	73 (56)	145 (53)	0.495
** CRT/CRT-D/ICD**	70 (60)	155 (61)	0.895
** Open heart surgery**	31 (24)	79 (28)	0.407
** Atrial fibrillation**	39 (30)	82 (29)	0.885
**Follow-up time, days (median (IQR))**	649 (740)	799 (678)	0.046
**Inclusion 2010-01-01, n (%)**	22 (17)	71 (25)	0.065
**Physical examination**			
** Heart rate, bpm**	82 ± (22)	79 ± (20)	0.114
** Systolic BP, mmHg**	127 ± (19)	130 ± (20)	0.076
** Diastolic BP, mmHg**	75 ± (13)	74 ± (12)	0.572
** BMI, n (%)**	28 ± (5)	27 ± (5)	0.131
** NT-proBNP (ng/L) (median (IQR))**	3140 (1338–8224)	3120 (1280–7448)	0.683
** P-haemoglobin, mmol/L**	133 ± (20)	128 ± (17)	0.003
** P-Sodium, mmol/L**	140 ± (3)	140 ± (3)	0.344
** P-Potassium, mmol/L**	4.3 ± (0.4)	4.3 ± (0.5)	0.448
**Index eGFR, ml/min/1.73m^2^**	48 ± (9)	41 ± (13)	<0.001
**CKD 3, n (%)**	127 (97)	225 (79)	<0.001
**CKD 4, n (%)**	2 (2)	51 (18)	<0.001
**CKD 5, n (%)**	2 (2)	8 (3)	0.273
**Medications, n (%)**			
** ACEI/ARB**	111 (85)	229 (80)	0.283
** Beta-blocker**	114 (87)	226 (79)	0.058
** Loop diuretic**	106 (92)	189 (77)	0.001
** Thiazide diuretic**	4 (6)	14 (8)	0.483

ACEI, angiotensin-converting enzyme inhibitor; ARB, angiotensin receptor blocker; MRA, mineralocorticoid receptor antagonist; EF, ejection fraction; CRT, Cardiac Resynchronization Therapy; CRT-D, CRT with defibrillator; ICD, Implantable Cardioverter-Defibrillator; BP, blood pressure; BMI, body mass index; NT-proBNP, N-terminal pro–B-type natriuretic peptide; eGFR, estimated Glomerular Filtration Rate; CKD, chronic kidney disease; RAAS-I, Renin-Angiotensin-Aldosterone System Inhibitor; BB, beta blockade. a) Values are means and standard deviation (SD), no. (%), or median (interquartile range (IQR)) when appropriate. *P* values are from the *X*^2^, Student ***t*** test, Mann Whitney U-Test or Fishers exact test as appropriate b) Coronary artery disease defined as either previous myocardial infarction or documented stenosis of ≥ 50%. c) Open heart surgery includes CABG/heart valve surgery/other.

### Effect of MRA on renal function

**Fig 2:**
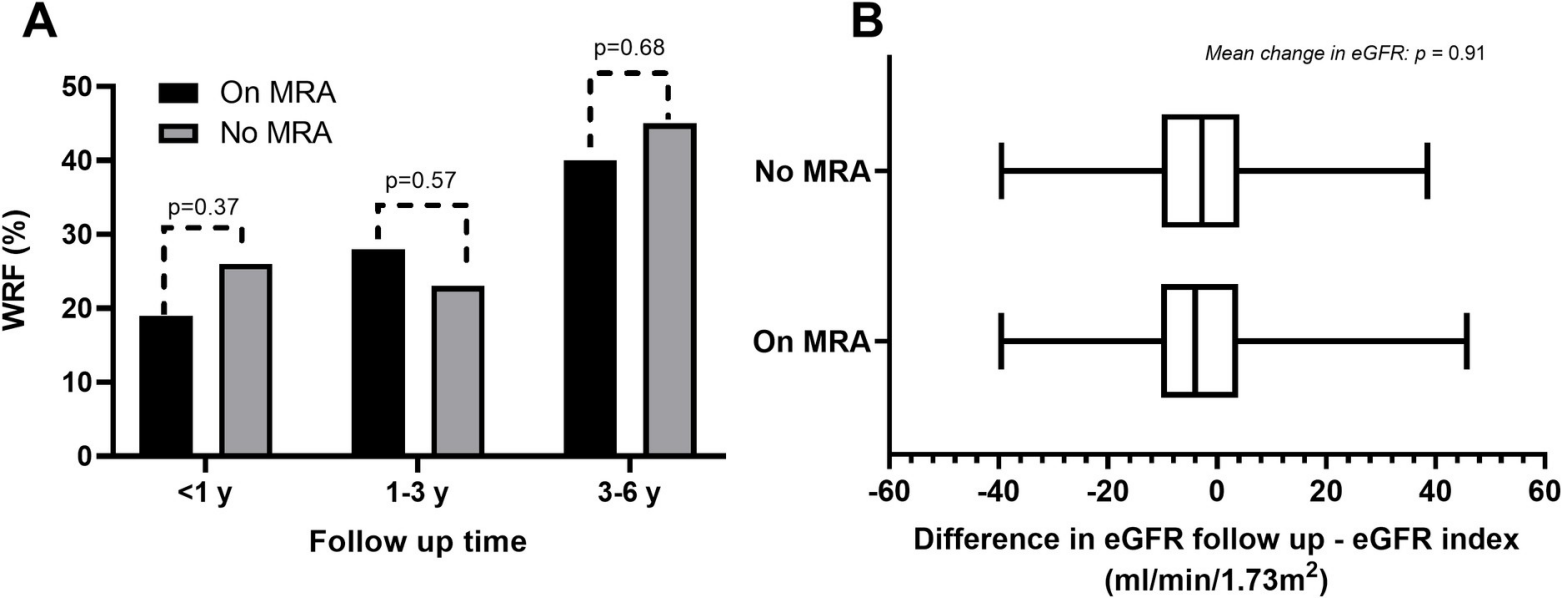
A) Frequency of worsening renal function (WRF) stratified into follow-up time. B) Change in eGFR between index and follow-up.

The On MRA group had a higher index eGFR compared to the No MRA group (48 vs 41 ml/min/1.73 m^2^ p<0.001). Overall, 128 patients (32%) experienced WRF, 32 patients (25%) in On MRA and 83 patients (30%) in No MRA (p = 0.293). When stratified in follow-up time, WRF was more common with longer follow-up, but with no difference between the groups. (Fig 2A). During follow-up, both groups experienced similar decline in mean eGFR (ml/min/1.73 m^2^), with -0.86 (±14 S.D.) in On MRA and with -0.47 (±14 S.D.) in No MRA (p = 0.87) (Fig 2B).

Serum-potassium (S-potassium) increased by a mean of 0.1 mmol/L in the On MRA group compared decreased by a mean of to -0.02 in the No MRA group (p = 0.057) and there was no difference on serious hyperkalemia (s-potassium >6.0 mmol/L) between the On MRA compared to No MRA (3 (2%) vs 2 (0.7%), p = 0.183). At index, there was no difference in patients with moderate hyperkalemia (S-potassium >5mmol/L) between On MRA and No MRA (n = 6 (5%) vs n = 19 (6%), p = 0.383). At follow-up, 10 patients On MRA (8%) had at least moderate hyperkalemia and 15 patients in No MRA (6%) (p = 0.349).

Follow-up eGFR was missing for 12 (3%) patients, why a total of 404 patients were included in the final analysis on decline in renal function. In multivariable analysis, hospitalization for heart failure and systolic blood pressure at index were associated with WRF. Noteworthy, use of MRA was not associated with WRF (Table 2). Finally, 50 patients discontinued MRA during follow-up of whom 20 (40%) had WRF.

**Table 2:** Factors associated with worsening renal function.

Factor	OR (95% CI)	*p*
**MRA**	0.81 (0.48–1.35)	0.421
**Age**	1.02 (1.00–1.05)	0.100
**Female Sex**	0.65 (0.41–1.03)	0.069
**eGFR index**	1.01 (0.99–1.03)	0.285
**Diabetes**	1.11 (0.67–1.84)	0.673
**SBT at index**	1.01 (1.00–1.03)	<0.015
**Hospitalization for HF**	2.13 (1.34–3.39)	<0.001

OR, odds ratio; CI, confidence interval; MRA, mineralocorticoid receptor antagonist; eGFR, estimated Glomerular Filtration Rate; SBT, systolic blood pressure; HF, heart failure. WRF is eGFR ≥20% between index and follow-up. The OR and 95% CI are adjusted logistic regression.

### Influence of MRA and renal function on survival

**Fig 3:**
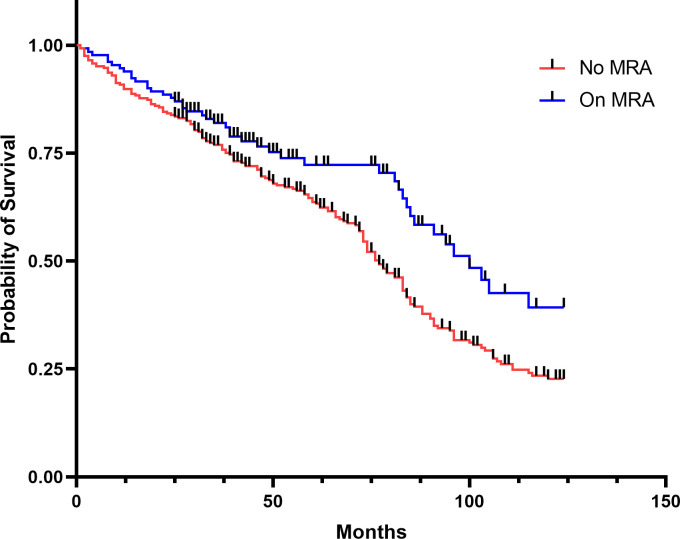
Kaplan-Meier survival plot for On MRA versus No MRA. Log rank p<0.001.

The On MRA group compared to the No MRA group had a higher probability of survival, log rank p<0.001 (Fig 3). In total there were 221 deaths (53%). 45 (34%) in On MRA and 176 (62%) in No MRA deceased during the study time (p<0.001).

Use of MRA at index and follow-up did not increase the risk of all-cause mortality, when adjusted for covariates (HR 0.93; 95% CI, 0.66–1.32 p = 0.685). Furthermore, higher index eGFR lowered the risk of all-cause mortality (HR 0.97; 95% CI 0.96–0.98; p<0.001) (Table 3). Worsening renal function, defined as >20% decline in eGFR, was associated with increased all-cause mortality when adjusted for covariates (HR 1.43; 95% CI, 1.07–1.89 p = 0.014) (Table 3).

**Table 3:** Adjusted risk of all cause-mortality in HFrEF patients with moderately impaired renal function.

Factors	B	*p*	HR	Lower 95% CI	Upper 95% CI
**Female Sex**	0.032	0.827	1.032	0.78	1.37
**MRA**	-0.273	0.685	0.930	0.66	1.32
**WRF**	0.354	0.014	1.425	1.07	1.89
**Age**	0.027	<0.001	1.027	1.01	1.04
**eGFR index**	-0.028	<0.001	0.973	0.96	0.98

HR, hazard ratio; CI, confidence interval; MRA, mineralocorticoid receptor antagonist; eGFR, estimated Glomerular Filtration Rate; WRF is eGFR ≥20% between index and follow-up. The OR and 95% CI are adjusted logistic regression.

When only including patients with WRF, there was 78 deaths (68%) in the 115 patients with WRF, to be compare with 138 deaths (48%) for the 289 patients without WRF (p<0.001). Nevertheless, use of MRA at index and follow-up did not increase the adjusted overall risk of mortality even when experiencing WRF (HR 1.20; 95% CI, 0.64–2.26 p = 0.567) (S1 Table).

## Discussion

Patients with HFrEF and reduced kidney function had a mortality rate of more than 53% with a median follow-up time of 2 years in our study. Use of MRA was not associated with decline in eGFR or worsening renal function. Moreover, MRA was not associated with adjusted all-cause mortality in HFrEF patients with moderately reduced index eGFR. A third of all patients developed WRF, regardless of treatment with MRA. Furthermore, in patients developing WRF, MRA was not associated with adjusted all-cause mortality.

With an initial eGFR < 60 ml/min/1.73 m^2^, eGFR declined by a similar rate regardless of MRA use. Although eGFR declined by 20% (WRF) in about a third of all patients, use of MRA did not increase the risk of WRF. The mean decline in eGFR did not decrease below eGFR 30 ml/min/1.73 m^2^. If the patients that discontinued MRA would be added to the On MRA group, the percentage of patients experience WRF would be slightly higher, 34% instead of 32%. Unfortunately, data on eGFR at discontinuation was not available. Patients in the On MRA group had a significantly higher index eGFR, which support previous findings that patients approximating an eGFR of 30 ml/min/1.73 m^2^ are undertreated with MRA [2, 14]. Perhaps, the low number of patients treated with MRA on the lower range of eGFR are caused by the clinical dilemma of applying results from clinical trials on an older and sicker real life population, causing the treating physician to hesitate to initiate MRA, especially in patients with impaired renal function [7, 9]. There was no difference in index s-potassium or change in s-potassium between the groups during the follow-up and the number of patients with moderate hyperkalemia was consistently low. However, since there are no available values between index and follow-up, the real incidence of hyperkalemia not could be extracted from the database.

WRF is an independent risk factor for mortality in patients with HFrEF [3, 6, 18]. While previous studies have shown that patients with MRA more often experience WRF, use of MRA was not associated with WRF in our study, even when stratified due to follow-up time [19, 20]. Notably, we had slightly higher background incidence of WRF in both groups than shown in previous studies, which could be explained by moderately impaired renal function at index and the high mean age [3, 6, 18]. The survival benefits associated with use of MRA in this study were due to higher index eGFR and younger age among patients treated with MRA. In large MRA trials such as RALES-HF, EPHASUS-HF, EMPHASIS-HF 17%, 17% and 27% developed WRF when put on MRA which in all trials were significantly higher than the control groups [19–21]. However, in all these studies, subgroup analyses showed that the overall benefit of MRA was present in patients with moderately reduced impaired function at inclusion.

Hospitalization for heart failure was associated with WRF. An increased risk of WRF within the first days of hospital admission has been demonstrated before, suggesting that decompensated heart failure combined with the impact of therapy administrated upon admission contributes to WRF [22]. Furthermore, higher index systolic blood pressure correlated with WRF. Hypertension has previously been seen to correlate with WRF in heart failure patients, and as many as 66% versus 70% of all patients in this study had a history of hypertension, which could explain this correlation [23].

Overall, about 48% of all HFrEF patients had moderately impaired renal function, defined as eGFR below 60 ml/min/1.73 m^2^ [6, 24]. Heart failure and chronic kidney disease (CKD) frequently coexists. Heart failure is a risk factor for developing CKD due to reduced perfusion and increased venous pressure, simultaneously CKD plays a role in the pathophysiology of heart failure [25]. Patients with estimated glomerular function (eGFR) <30 ml/min/1.73 m^2^ have generally been excluded from randomized clinical trials in fear of WRF and intolerance, causing lack of evidence for therapy with MRA in this group [10].

Despite this, previous studies shows that HFrEF patients benefit from Renin-angiotensin-aldosterone system (RAAS) inhibitors to a further extent if WRF is present, probably because of greater improvement of RAAS-blockade when RAAS is already overactivated [3, 15]. There is some evidence that treatment with MRA has a reno-protective effect as elevated plasma levels of aldosterone may contribute to worsening renal function by inducing endothelial dysfunction, left ventricular hypertrophy, and increased mortality [26, 27]. In this study of HFrEF patients with moderately impaired renal function we had a mean age of 80 years and a high frequency of comorbidities such as diabetes and hypertension. In patients with chronic kidney disease or diabetic nephropathy, MRA has been shown to reduce macroalbuminuria and lowering blood pressure [28]. Further, in patients with chronic kidney disease MRA seems to reduce proteinuria with a statistically significant, but clinically harmless, increase in potassium [29]. In patients with diabetes MRA have been showed to reduce albuminuria >30% with a reversible initial reduction in eGFR [30].

### Limitations

Observational data cannot definitively determine cause-and-effect relationships. This single-centre study design limits the generalizability and external validity of the results. On the other hand, the real-world heart failure population more accurately reflects patients with HFrEF and moderately impaired renal function as our patients are older with more comorbidities that usually are exclusion criteria in many randomized controlled trials.

We tried to compensate for the differences in follow-up time by stratify the outcomes of WRF into follow-up time to determine how it affected the results. Since this study is This was a retrospective, observational study it is inherited an uncertainty of events between index and follow-up. For example, we could not calculate the incidence of hyperkalemia by only two s-potassium values from index and follow-up. According to guidelines and clinical praxis, treatment with MRA requires treatment with ACEI/ARB and BB in HFrEF why the correlation between these drugs inhibit inclusion of RAAS-I or BB in the multivariable analysis [31]. On the other hand, patients were equally distributed by treatment with ACEI/ARB and BB. Unfortunately, the data in the medical records did not include information enough to assess New York Heart Association (NYHA) function class. Further, more research is needed on the patients that discontinued MRA.

## Conclusions

We studied a real-world heart failure population with moderately reduced kidney function. This group of patients had an overall high mortality rate and WRF were common regardless of treatment with MRA or not. There were no signs of detrimental effects from MRA treatment on survival or worsening renal function.

## Supporting information

S1 Table All-cause mortality in HFrEF patients with moderately impaired renal function experience WRF during follow-up. MRA, mineralocorticoid receptor antagonist; eGFR, estimated Glomerular Filtration Rate; WRF, Worsening Renal Function. WRF is eGFR >20% between index and follow-up. eGFR is calculated by the revised Lund-Malmö equation form S-Creatinine.
(DOCX)

## References

[1] Greene SJ, Butler J, Albert NM, DeVore AD, Sharma PP, Duffy CI, et al. Medical Therapy for Heart Failure With Reduced Ejection Fraction: The CHAMP-HF Registry. J Am Coll Cardiol. 2018;72(4):351–66. doi: 10.1016/j.jacc.2018.04.070 30025570

[2] Savarese G, Carrero JJ, Pitt B, Anker SD, Rosano GMC, Dahlstrom U, et al. Factors associated with underuse of mineralocorticoid receptor antagonists in heart failure with reduced ejection fraction: an analysis of 11 215 patients from the Swedish Heart Failure Registry. Eur J Heart Fail. 2018;20(9):1326–34. doi: 10.1002/ejhf.1182 29578280

[3] Clark H, Krum H, Hopper I. Worsening renal function during renin-angiotensin-aldosterone system inhibitor initiation and long-term outcomes in patients with left ventricular systolic dysfunction. European journal of heart failure. 2014;16(1):41–8. doi: 10.1002/ejhf.13 24453097

[4] Metra M, Nodari S, Parrinello G, Bordonali T, Bugatti S, Danesi R, et al. Worsening renal function in patients hospitalised for acute heart failure: clinical implications and prognostic significance. Eur J Heart Fail. 2008;10(2):188–95. doi: 10.1016/j.ejheart.2008.01.011 18279773

[5] Damman K, Jaarsma T, Voors AA, Navis G, Hillege HL, van Veldhuisen DJ. Both in- and out-hospital worsening of renal function predict outcome in patients with heart failure: results from the Coordinating Study Evaluating Outcome of Advising and Counseling in Heart Failure (COACH). European journal of heart failure. 2009;11(9):847–54. doi: 10.1093/eurjhf/hfp108 19696057

[6] de Silva R, Nikitin NP, Witte KK, Rigby AS, Goode K, Bhandari S, et al. Incidence of renal dysfunction over 6 months in patients with chronic heart failure due to left ventricular systolic dysfunction: contributing factors and relationship to prognosis. Eur Heart J. 2006;27(5):569–81. doi: 10.1093/eurheartj/ehi696 16364971

[7] Pitt B, Zannad F, Remme WJ, Cody R, Castaigne A, Perez A, et al. The effect of spironolactone on morbidity and mortality in patients with severe heart failure. Randomized Aldactone Evaluation Study Investigators. N Engl J Med. 1999;341(10):709–17. doi: 10.1056/NEJM199909023411001 10471456

[8] Zannad F, McMurray JJ, Krum H, van Veldhuisen DJ, Swedberg K, Shi H, et al. Eplerenone in patients with systolic heart failure and mild symptoms. N Engl J Med. 2011;364(1):11–21. doi: 10.1056/NEJMoa1009492 21073363

[9] Pitt B, Remme W, Zannad F, Neaton J, Martinez F, Roniker B, et al. Eplerenone, a selective aldosterone blocker, in patients with left ventricular dysfunction after myocardial infarction. N Engl J Med. 2003;348(14):1309–21. doi: 10.1056/NEJMoa030207 12668699

[10] McDonagh TA, Metra M, Adamo M, Gardner RS, Baumbach A, Böhm M, et al. 2021 ESC Guidelines for the diagnosis and treatment of acute and chronic heart failure. Eur Heart J. 2021. 10.1093/eurheartj/ehab853 34922348

[11] Heywood JT, Fonarow GC, Yancy CW, Albert NM, Curtis AB, Gheorghiade M, et al. Comparison of medical therapy dosing in outpatients cared for in cardiology practices with heart failure and reduced ejection fraction with and without device therapy: report from IMPROVE HF. Circulation Heart failure. 2010;3(5):596–605. doi: 10.1161/CIRCHEARTFAILURE.109.912683 20634483

[12] Thorvaldsen T, Benson L, Dahlstrom U, Edner M, Lund LH. Use of evidence-based therapy and survival in heart failure in Sweden 2003–2012. European journal of heart failure. 2016;18(5):503–11. doi: 10.1002/ejhf.496 26869252

[13] Maggioni AP, Dahlstrom U, Filippatos G, Chioncel O, Crespo Leiro M, Drozdz J, et al. EURObservational Research Programme: regional differences and 1-year follow-up results of the Heart Failure Pilot Survey (ESC-HF Pilot). Eur J Heart Fail. 2013;15(7):808–17. doi: 10.1093/eurjhf/hft050 23537547

[14] Jonsson A, Norberg H, Bergdahl E, Lindmark K. Obstacles to mineralocorticoid receptor antagonists in a community-based heart failure population. Cardiovasc Ther. 2018;36(5):e12459. doi: 10.1111/1755-5922.12459 30019390 PMC6175311

[15] Peterson PN, Rumsfeld JS, Liang L, Hernandez AF, Peterson ED, Fonarow GC, et al. Treatment and risk in heart failure: gaps in evidence or quality? Circ Cardiovasc Qual Outcomes. 2010;3(3):309–15. doi: 10.1161/CIRCOUTCOMES.109.879478 20388872

[16] Levey AS, Eckardt KU, Tsukamoto Y, Levin A, Coresh J, Rossert J, et al. Definition and classification of chronic kidney disease: a position statement from Kidney Disease: Improving Global Outcomes (KDIGO). Kidney Int. 2005;67(6):2089–100. doi: 10.1111/j.1523-1755.2005.00365.x 15882252

[17] Jonsson A, Viklund I, Valham F, Bergdahl E, Lindmark K, Norberg H. Comparison of creatinine-based methods for estimating glomerular filtration rate in patients with heart failure. ESC Heart Fail. 2020;7(3):1150–60. doi: 10.1002/ehf2.12643 32052932 PMC7261582

[18] Damman K, Valente MA, Voors AA, O’Connor CM, van Veldhuisen DJ, Hillege HL. Renal impairment, worsening renal function, and outcome in patients with heart failure: an updated meta-analysis. Eur Heart J. 2014;35(7):455–69. doi: 10.1093/eurheartj/eht386 24164864

[19] Vardeny O, Wu DH, Desai A, Rossignol P, Zannad F, Pitt B, et al. Influence of baseline and worsening renal function on efficacy of spironolactone in patients With severe heart failure: insights from RALES (Randomized Aldactone Evaluation Study). Journal of the American College of Cardiology. 2012;60(20):2082–9. doi: 10.1016/j.jacc.2012.07.048 23083787

[20] Rossignol P, Cleland JG, Bhandari S, Tala S, Gustafsson F, Fay R, et al. Determinants and consequences of renal function variations with aldosterone blocker therapy in heart failure patients after myocardial infarction: insights from the Eplerenone Post-Acute Myocardial Infarction Heart Failure Efficacy and Survival Study. Circulation. 2012;125(2):271–9. doi: 10.1161/CIRCULATIONAHA.111.028282 22128223

[21] Rossignol P, Dobre D, McMurray JJ, Swedberg K, Krum H, van Veldhuisen DJ, et al. Incidence, determinants, and prognostic significance of hyperkalemia and worsening renal function in patients with heart failure receiving the mineralocorticoid receptor antagonist eplerenone or placebo in addition to optimal medical therapy: results from the Eplerenone in Mild Patients Hospitalization and Survival Study in Heart Failure (EMPHASIS-HF). Circ Heart Fail. 2014;7(1):51–8. doi: 10.1161/CIRCHEARTFAILURE.113.000792 24297687

[22] Forman DE, Butler J, Wang Y, Abraham WT, O’Connor CM, Gottlieb SS, et al. Incidence, predictors at admission, and impact of worsening renal function among patients hospitalized with heart failure. Journal of the American College of Cardiology. 2004;43(1):61–7. doi: 10.1016/j.jacc.2003.07.031 14715185

[23] Krumholz HM, Chen YT, Vaccarino V, Wang Y, Radford MJ, Bradford WD, et al. Correlates and impact on outcomes of worsening renal function in patients > or = 65 years of age with heart failure. Am J Cardiol. 2000;85(9):1110–3. doi: 10.1016/s0002-9149(00)00705-0 10781761

[24] Szummer K, Evans M, Carrero JJ, Alehagen U, Dahlstrom U, Benson L, et al. Comparison of the Chronic Kidney Disease Epidemiology Collaboration, the Modification of Diet in Renal Disease study and the Cockcroft-Gault equation in patients with heart failure. Open Heart. 2017;4(2):e000568. doi: 10.1136/openhrt-2016-000568 28761677 PMC5515135

[25] Damman K, Voors AA, Navis G, van Veldhuisen DJ, Hillege HL. The cardiorenal syndrome in heart failure. Prog Cardiovasc Dis. 2011;54(2):144–53. doi: 10.1016/j.pcad.2011.01.003 21875513

[26] Brown NJ. Eplerenone: cardiovascular protection. Circulation. 2003;107(19):2512–8. doi: 10.1161/01.CIR.0000071081.35693.9A 12756192

[27] Hollenberg NK. Aldosterone in the development and progression of renal injury. Kidney Int. 2004;66(1):1–9. doi: 10.1111/j.1523-1755.2004.00701.x 15200407

[28] Sarafidis PA, Memmos E, Alexandrou ME, Papagianni A. Mineralocorticoid Receptor Antagonists for Nephroprotection: Current Evidence and Future Perspectives. Curr Pharm Des. 2018;24(46):5528–36. doi: 10.2174/1381612825666190306162658 30848187

[29] Bianchi S, Bigazzi R, Campese VM. Antagonists of aldosterone and proteinuria in patients with CKD: an uncontrolled pilot study. American journal of kidney diseases: the official journal of the National Kidney Foundation. 2005;46(1):45–51. doi: 10.1053/j.ajkd.2005.03.007 15983956

[30] Schjoedt KJ, Rossing K, Juhl TR, Boomsma F, Rossing P, Tarnow L, et al. Beneficial impact of spironolactone in diabetic nephropathy. Kidney Int. 2005;68(6):2829–36. doi: 10.1111/j.1523-1755.2005.00756.x 16316360

[31] Ponikowski P, Voors AA, Anker SD, Bueno H, Cleland JGF, Coats AJS, et al. 2016 ESC Guidelines for the diagnosis and treatment of acute and chronic heart failureThe Task Force for the diagnosis and treatment of acute and chronic heart failure of the European Society of Cardiology (ESC)Developed with the special contribution of the Heart Failure Association (HFA) of the ESC. European Heart Journal. 2016;37(27):2129–200. doi: 10.1093/eurheartj/ehw128 27206819

