# Supplementary material for: Mineralocorticoid receptor antagonists use in patients with heart failure and impaired renal function
Source: PLoS One. 2021 Oct 28;16(10):e0258949. doi: 10.1371/journal.pone.0258949 (PMC8553049; doi:10.1371/journal.pone.0258949)
Supplement: S1 Table — MRA, mineralocorticoid receptor antagonist; eGFR, estimated Glomerular Filtration Rate; WRF, Worsening Renal Function. WRF is eGFR >20% between index and follow-up. eGFR is calculated by the revised Lund-Malmö equation form S-Creatinine. (DOCX) [file pone.0258949.s001.docx]

# Supplementary materials

S1 Table. All-cause mortality in HFrEF patients with moderately reduced renal function experience WRF during follow-up

|  | B | *p* | HR | Lower 95% CI | Upper 95% CI |
| --- | --- | --- | --- | --- | --- |
| Female Sex | -0.163 | 0.474 | 0.848 | 0.54 | 1.33 |
| Age | 0.018 | 0.211 | 1.019 | 0.99 | 1.05 |
| On MRA | 0.184 | 0.567 | 1.202 | 0.64 | 2.26 |
| eGFR index | -0.030 | 0.005 | 0.970 | 0.95 | 0.99 |

MRA, mineralocorticoid receptor antagonist; eGFR, estimated Glomerular Filtration Rate; WRF, Worsening Renal Function. WRF is eGFR >20% between index and follow-up. eGFR is calculated by the revised Lund-Malmö equation form S-Creatinine.
